# Memory Functions in Recreational Pistol Sport Shooters: Does Lead Matter?

**DOI:** 10.4137/EHI.S894

**Published:** 2009-04-03

**Authors:** Sanna Asa-Mäkitaipale, Mervi Jehkonen, Jukka Uitti, Juhani Vilkki

**Affiliations:** 1 South Karelian Central Hospital, Department of Neurology, Lappeenranta, Finland; 2 University of Helsinki, Department of Psychology, Helsinki, Finland; 3 University of Tampere, Department of Psychology, Tampere, Finland; 4 Finnish Institute of Occupational Health, Tampere, Finland; 5 Clinic of Occupational Medicine, Tampere University Hospital, Tampere, Finland

**Keywords:** cognitive function, lead exposure, sport shooting

## Abstract

**Objective:**

The aim of our study was to examine the memory functions of pistol sport shooters using powder charges when exposure to lead is expected to be considerably lower than in occupational circumstances.

**Methods:**

A neuropsychological battery of memory and intelligence tests was administered to 20 sport shooters and 20 controls whose mean ages (SDs) were 55 (9.6) and 54 (9.3) years respectively. Memory functions were evaluated with three subtests of the Wechsler Memory Scale - Revised (WMS-R) and an incidental memory test. Intelligence was assessed with four subtests of the Wechsler Adult Intelligence Scale - Revised (WAIS-R). The level of alcohol consumption and depression were examined in both groups. Blood lead level was determined among the shooters.

**Results:**

The shooters performed worse than the controls in the tests of incidental and logical memory. The groups did not differ in intelligence, mood or alcohol consumption. The mean (SD) blood lead level of the sport shooters was 0.52 μmol/L (0.40), responding 10.76 μg/dl (8.28).

**Conclusions:**

Low lead exposure in recreational shooting conditions may impair verbal memory. Therefore it is important to ensure that lead exposure is prevented among those shooting for sport.

## Introduction

Shooting with lead-containing bullets on indoor firing ranges is known to expose people to lead.^1^–^3^ On being fired an unjacketed lead bullet emits some lead fumes and dust which become airborne due to the gun’s muzzle blast.^4^,^5^ The primary exposure of lead occurs when the shooter inhales the fumes in the proximity of the gun.^6^ Lead exposure in indoor firing ranges can also occur through inhalation of lead fumes and particulates suspended in the range air. The lead exposure is also dependent on the structure of the indoor range and the ventilation systems.^5^ Lead exposure can be examined in blood lead level, which is known to be elevated among trainees, instructors, police officers and controllers^1^–^3^,^5^,^6^ and among recreational shooters.^7^,^8^ Lead exposure is more intensive in closed shooting ranges than in open ranges, where shooting is normally not considered to be hazardous to sport shooters’ health.^5^

Numerous studies have demonstrated that occupational lead exposure is associated with low neuropsychological test performance.^9^–^23^ Long-term environmental exposure to high levels of lead has persistent detrimental effects on cognitive functions in older adults.^24^ High lead exposure may damage the central nervous system.^25^ High tibial lead is associated with small total brain volume.^26^,^27^ Lead may influence cognitive functions by decreasing of brain volume.^28^

Lead may affect memory, executive functions, reasoning, visual and motor functions.^12^–^20^,^21^ Memory functions are known to be vulnerable, not least to lead exposure which may have effects both on verbal and visual memory.^14^,^18^–^20^ Lead may impair verbal memory even at occupational exposures and have progressive effects later when exposure has finished.^20^ Chronic lead exposure affects encoding, storage and retrieval of verbal information.^23^ Lead may impair the ability to remember large verbal entities,^14^,^18^ verbal short-term or long-term memory.^19^,^20^,^23^ In visual memory, the effects of lead exposure can be seen later, with long-term effects.^20^ The effects of lead have been found in visual retention memory.^18^,^20^ So far it has not been demonstrated at what level of lead in the blood the cognitive functions begin to be impaired in adults.^29^ In children blood lead concentrations below 10 μg/dl (responding 0.48 μmol/l) are associated with impaired intelligence,^30^–^32^ even as low blood lead levels as 7.5 ug/dl (responding 0.36 μmol/l) have been found to decrease children’s intellectual functions because of environmental exposure.^33^

The aim of this study was to examine sport shooters’ memory functions since lead exposure is known to affect memory. To the best of our knowledge there is very little evidence of the neuropsychological effects caused by lead exposure in sport shooters using powder charges. We wanted to examine if lead impairs the memory functions of sport shooters as evaluated in clinical neuropsychological memory tests. We assumed that sport shooters would perform worse than controls in memory tests as a result of lead exposure.

## Participants and Methods

### Participants

The study included 20 sport shooters and 20 controls. The study population consisted of pistol sport shooters who practised on indoor or outdoor firing ranges, mainly with unjacketed lead bullets. All sport shooters from two local shooting clubs who volunteered to participate in the study were contacted. The sport shooters had to meet the following inclusion criteria: a) at least 10 years of continuous sport shooting with lead bullets in indoor or outdoor shooting ranges, and b) male gender. The control group consisted of volunteers, who were similar to the shooters regarding to gender, age and years of education. The controls did not have any known lead exposure. The eligibility of respondents was confirmed by a subsequent questionnaire. The potential participants were excluded if any of the following criteria was met: a) neurological and/or psychiatric disease, b) alcohol or drug abuse, or c) other exposure to heavy metals and/or solvents.

### Methods

Each eligible respondent participated in a single two-hour neuropsychological examination. The participants completed questionnaires on personal background, alcohol consumption and shooting habits. The analysis of blood lead level was carried out only for the sport shooters.

#### Neuropsychological examination

A trained examiner blind to participants’ exposure administered neuropsychological tests which have been found to be useful in occupational studies. The clinical tests are shown in Table 1. Reasoning was examined with the Similarities, Digit Symbol, Block Design and Picture Completion subtests of the Wechsler Adult Intelligence Scale—Revised^34^ (WAIS-R). Memory was tested with the Logical Memory (Story A), Verbal Learning and Visual Retention subtests of the Wechsler Memory Scale - Revised^35^ (WMS-R), with an incidental memory test^36^ and with the Digit Span subtest of the WAIS-R.^34^

In the incidental memory test^36^ the Similarities Subtest of the WAIS-R was used as an orienting task. After the participant had answered the first 10 questions, in which he tried to discover the similarity between two words, he was asked, without previous warning, to recall the word pairs or words included in the questions in any order. After the free recall, the first word of each pair in which the participant did not recall both of the words in the free recall was given as a cue for the recall of the second word. The total number of correct words in the free and cued recall formed the score.

The level of depression was assessed with the Beck Depression Inventory^37^ (BDI). The range of its total score was 0–63. Alcohol consumption was examined by the Alcohol Use Disorders Identification Test^38^ (AUDIT) ranging from 0 to 40.

#### Analysis of blood lead level

Sport shooters participated in a blood lead (PbB) analysis. Blood samples were collected one week after the neuropsychological examination. An amount of 5–10 mL of whole blood in heparinised vacuum tube for trace element analysis was collected. Blood lead concentration was determined with an electrothermal atomic absortion spectrophotometric method^39^ in the biomonitoring laboratory of the Finnish Institute of Occupational Health.

#### Data analysis

Statistical Package for the Social Sciences (SPSS-PC version 11.5) was used for the data analysis. In the background variables (age, years of education, the BDI and the AUDIT) the group differences were studied with t-tests for independent groups. In the intelligence tests and in the immediate memory tests, the sums of raw scores were used, but in the delayed recalls of the memory tests percent variables. In the tests of logical memory and visual retention the percent variable was the delayed recall score as a percentage of the immediate recall score. In the verbal learning test the delayed recall score as a percentage of the third (last) immediate recall score was used. The difference between the two groups was tested with a multivariate analysis of covariance (MANCOVA) separately on the four intelligence and the nine memory variables, with age as a covariate. Correlations between the psychological test variables, the blood lead level and shooting habits were studied with Pearson’s correlation coefficients. The significance level of statistical analysis was set at p ≤ 0.05.

## Results

The sport shooters did not differ from the controls on the background variables (Table 2). The sport shooters had spent an average of seven hours on indoor or outdoor firing ranges during the last month. The range of years of sport shooting was 11 to 46 (mean = 27.4, SD = 11.6).

The results of the blood lead analysis and the amount of bullets fired during the last month are given in Table 3.

The mean blood lead level of the sport shooters was 0.52 μmol/L (responding 10.76 μg/dl), which is higher than the normal level 0.3 μmol/L (responding 6.21 μg/dl) of non-exposed people.^40^,^41^ The shooters’ blood lead level was higher the more bullets they had fired during the last month or during the last year (r = 0.705, p = 0.001; r = 0.551, p = 0.012).

The results of the neuropsychological tests are shown in Table 4. The difference between the two groups on the memory variables was significant [MANCOVA F(9,29) = 5.021, p ≤ 0.001]. The sport shooters’ performance was markedly poorer than that of the controls on the incidental memory test and on the immediate and delayed verbal logical memory. The groups did not differ significantly on the intelligence tests [MANCOVA F(4,34) = 1.577, p = 0.203]. The correlations between the psychological test scores and the blood lead level or the shooting habits did not reach statistical significance.

## Discussion

The aim of this study was to find whether sport shooters’ lead exposure has an adverse effect on memory functions. The sport shooters performed significantly worse than the controls on two verbal memory tests, namely on the immediate and delayed Logical Memory test and the incidental memory test. No other significant differences were found in the verbal or visual memory tests. The groups did not differ on the intelligence tests, on mood or alcohol consumption. To the best of our knowledge there is very little evidence so far about neuropsychological effects caused by lead exposure on firing ranges.

In our study the blood lead levels among sport shooters were slightly higher than the reference limit for non-exposed people. In Finland the reference level of non-exposed people is 0.3 μmol/L (responding 6.21 μg/dl) determined by Finnish Institute of Occupational Health. The level is in balance with other papers.^40^,^41^ The shooters’ mean blood lead level was lower than that found in earlier studies concerning shooting with lead bullets^2^,^6^,^7^ but still higher than that found among Swedish police officers.^3^ In spite of the low average blood lead level in this study, a significant correlation was found between the numbers of bullets fired during the last month or year and the blood lead levels of the sport shooters. This may indicate that the amount lead fumes inhaled in the proximity of the gun in firing ranges mainly increases the PbB level. This observation is supported by Demmeler et al,^8^ where rounds shot each month highly correlated with PbB concentrations of 129 recreational indoor-shooters. In our study it was assumed that the PbB levels of the controls did not exceed the reference limit for non-exposed people.

In studies reporting the detrimental effects of occupational lead exposure on verbal learning,^16^,^42^ the PbB levels were considerably higher than those of the present study. Peak tibial lead has been associated with poor performance in verbal memory.^19^ On the other hand, chronic occupational lead exposure, irrespective of the current PbB level, has been demonstrated to impair encoding and retrieval of verbal information in the Rey Auditory Verbal Learning Test^19^,^20^,^23^ and in the Logical Memory subtest of the WMS-R.^14^ Similar verbal memory deficits have been reported with cumulative environmental lead exposure irrespective of recent PbB levels.^24^ We did not examine the cumulative lead exposure, which would be an interesting topic for further studies. In the present study we focused on the current effects of continuous lead exposure on contemporary memory functions. According to our results, low recreational lead exposure is associated with impaired retrieval of verbal material in sport shooters. The groups did not differ in the Verbal Learning subtest of the WMS-R, which may indicate that encoding is not impaired in sport shooters and that the cueing in this test created an advantageous context to them for the retrieval of verbal associations.

There are contradictory results concerning the effect of low lead exposure on cognitive functions. On the one hand, relatively low blood lead levels (mean = 30.8 μg/100 ml; responding 1.49 μmol/l) due to occupational exposure have been associated with neuropsychological deficits, for example with executive and visuospatial dysfunctions.^21^ On the other hand, it has been documented that very low blood lead levels (mean = 5.4 μg/100 ml; responding 0.26 μmol/l) do not result in cognitive deficits in individuals who have formerly been occupationally exposed to lead.^43^ Interestingly, our study indicated that even a low, slightly elevated blood lead level is associated with impaired verbal memory after long-term exposure to low lead levels. The memory dysfunction was not caused by deficient verbal reasoning, because the performance of the sport shooters and the controls on the reasoning tests was almost equal. In our study the lead levels of the sport shooters were considerably lower than in other studies, which have documented neuropsychological defects caused by occupational lead exposure.^42^

One limitation of this study is that it was not possible to conduct the blood lead level analysis for the control group, although it is highly likely that the lead exposure of the controls did not exceed the normal level. It is worth noting that all sport shooters had practised shooting for many years. Although the blood lead level does not indicate the effect of long-term cumulative lead exposure, our results suggest that long-term recreational lead exposure results in memory impairment. To summarize, recreational lead exposure was associated with verbal memory deficits examined in clinical neuropsychological tests. In future studies it would be interesting to examine whether recreational lead exposure has equally progressive effects on verbal or visual memory as occupational lead exposure after that exposure ceases.

## Figures and Tables

**Table 1.** Neuropsychological tests.

**Cognitive domain**	**Tests**	**Possible range**
Verbal reasoning	Similarities^1^	0–34
Visual reasoning	Digit Symbol^1^	0–93
	Block Design^1^	0–51
	Picture Completion^1^	0–22
Verbal memory	Digit Span (a + b)^1^	0–28
	a) forward: 0–14	
	b) backward: 0–14	
	Logical Memory (Story A)^2^	0–25
	Verbal Learning^2^	0–24
	Incidental memory test^3^	0–20
Visual memory	Visual Retention^2^	0–41

**Notes:**

1 The subtests of the Wechsler Adult Intelligence Scale - Revised.

2 The subtests of the Wechsler Memory Scale - Revised.

3 The incidental memory test,^36^ see Methods.

**Table 2.** The characteristics of the participants.

	**Sport shooters**	**Controls**	**Comparison of groups**	
	**Mean [SD; Range]**	**Mean [SD; Range]**	**t-value**	**p**
Age (years)	55.0 [9.6; 30–68]	54.0 [9.3; 30–67]	0.44	0.67
Years of education	12.7 [3.6; 7–20]	13.3 [4.4; 8–23]	0.49	0.63
BDI	5.6 [4.7; 0–17]	5.1 [4.0; 0–12]	0.32	0.75
AUDIT	4.7 [3.4; 0–13]	5.7 [4.1; 0–13]	0.84	0.40

**Abbreviations:** SD, standard deviation; p, significance; BDI, Beck Depression Inventory;^37^ range 0–63, AUDIT, Alcohol Use Disorders Identification Test;^38^ range 0–30.

**Table 3.** The blood lead level and the number of shot bullets and their correlations.

	**Mean**	**SD**	**r**	**p**
Blood lead (μmol/l)	0.52	0.40		
Bullets fired during the last month	326.00	509.28	0.705	0.001
Bullets fired during the last year	4645.00	4908.37	0.551	0.012

**Abbreviations:** SD, standard deviation; r, Pearson correlation coefficient; p, significance.

**Table 4.** Comparison of the neuropsychological test scores between shooters and controls tested with MANCOVA.

**Neuropsychological test**	**Shooters**		**Controls**		**F-value**	**p**
	**Mean**	**SD**	**Mean**	**SD**		
**A. Memory tests (WMS-R)**						
Incidental memory^1^	13.85	1.93	15.60	1.39	13.71	≤0.001
Logical Memory, immediate recall	10.40	3.25	13.50	3.33	4.32	0.021
Logical Memory, delayed recall^2^	73.61	11.05	84.80	11.69	5.06	0.011
Verbal Learning, immediate recall	14.60	4.54	15.35	4.34	0.16	0.689
Verbal Learning, delayed recall^2^	101.52	27.69	105.08	23.58	0.14	0.713
Digit Span, forward	7.20	0.37	8.00	0.38	2.84	0.071
Digit Span, backward	6.95	0.33	6.55	0.37	0.84	0.620
Visual Retention, immediate recall	34.95	3.49	33.20	4.98	2.82	0.072
Visual Retention, delayed recall^2^	89.26	14.59	90.36	21.24	1.61	0.215
**B. Intelligence tests (WAIS-R)**						
Similarities	28.25	2.92	28.65	3.27	0.16	0.691
Picture Completion	18.10	1.83	18.45	2.01	0.25	0.624
Block Design	33.70	8.70	32.05	9.93	0.84	0.365
Digit Symbol	44.55	9.67	48.25	10.54	1.12	0.298

**Abbreviations:** SD, standard deviation; p, significance; WMS-R, Wechsler Memory Scale - Revised; see possible ranges in Table 1,

1 Vilkki et al. 1992,

2 See Methods; WAIS-R, Wechsler Adult Intelligence Scale - Revised; see possible ranges in Table 1.
